# 

**DOI:** 10.1038/sj.bjc.6600403

**Published:** 2002-01-02


**S1**
**REDESIGNING CANCER THERAPY**
Professor Sir David P. Lane FRS. Dept of Surgery and Molecular Oncology Dundee University.

The completion of the human genome project and a rapidly enhanced understanding of the molecular mechanism of human disease are creating a wealth of new targets for drug discovery. Keeping pace with this are the development of novel technologies that can enhance the speed and accuracy of drug discovery at every level of the process. Importantly these technologies have a great impact on the cost of drug development enabling academic and small Biotech companies to make larger contributions to the process. Conversely the recognition of molecular variation in the disease process and in the metabolic and toxicological response of individuals to therapeutic drugs implies the individualisation of treatment and care that may favour the development of a larger number of medicines to treat a particular disease. All of these processes are particularly clear in the development of new anti-cancer drugs. The Cancer Research Campaign in the UK and the NCI in the USA have both instituted schemes that allow academic groups access to diverse chemical libraries and high throughput screening against validated targets emerging from basic research. Target validation has been greatly aided by the development of reporter cell lines, knock out mouse derived cell lines and dominant negative mutant and anti-sense approaches. Commercially small biotech companies have access to large and diverse chemical libraries and success with in silico screening using molecular docking software to screen "virtual" libraries is greatly reducing the number of molecules that must be physically tested for activity. This critically depends on the increasing rapidity of 3D-structure determination. Exciting examples that have emerged from these new approaches include inhibitors and activators of the p53 response, signal transduction modifiers and pro-apoptotic agents. Downstream challenges include more rapid assessment of PK, metabolism and toxicology. The new drugs, tailored to specific molecular forms of disease, will also pose novel problems for the regulators, practitioner and patients.

**S2**
**CURRENT INDICATIONS & FUTURE STRATEGIES FOR ADJUVANT SYSTEMATIC THERAPY IN EARLY BREAST CANCER**
Dr Matti Aapro, Clinique de Genolier, Switzerland

ABSTRACT NOT RECEIVED

**S3**
**CLOSE ENCOUNTERS OF THE MOLECULAR KIND**
Malcolm Stevens, Cancer Research UK Experimental Cancer Chemotherapy Group, Cancer Research Laboratories, University of Nottingham NG7 2RD

With the possible exception of taxol and combrestestatin anticancer drugs don't grow on trees. Rather they have 'emerged', usually following the initial efforts of chemists, and have been subjected to chemical, pharmacological and pharmaceutical evolutionary forces which have weeded out the unfitted before they are subjected to the ultimate challenge – do they work in the clinic, or not? In the past some of the best agents have secured clinical respectability before even their molecular targets have been correctly identified. This approach is perceived by many to be out-dated and its practitioners facing, like the dinosaurs, an extinction event. In the post-genomics age the modern paradigm envisages drug discovery to be a more functional exercise: identification and validation of a molecular target; a hunt for new molecules synthesised by combinatorial chemistry; identification of 'hits' through high-throughput screening; a honing of lead structures through conventional medicinal chemistry; and selection of a clinical candidate. Sounds easy doesn't it? But now there is a whisper abroad that this process is proving cumbersome and impracticable, even before it has chalked up its first clinical success. And what may be perceived as a 'smart' molecular target today may be rendered irrelevant by fast moving biological frontiers tomorrow.

The most precious commodities in drug discovery cancer research, I will argue, are novel molecules with novel biological properties, irrespective of how they are identified – by high-throughput technologies, chemical inquisitiveness, or even trawling the oceans and combing the rain forests. Also there has to be a place on the drug discovery landscape for those with acute instincts for molecular inventiveness. The key skills required are centred on those Cinderella subjects – the chemical and physical sciences and pharmacology – but melded to an in-depth knowledge of breakthroughs in cancer biology. (Keeping up with the literature in all these areas is a treadmill indeed!) Such people will comfortably wear molecules like they might do their favourite shirt; their close encounters of the molecular kind will have been obtained through a long apprenticeship at the bench; their antennae will be attuned to be able to recognise what is a cunning (not necessarily stunning) molecule.
Tom Connors was such a person. If Cancer Research UK is to achieve its mission to discover and develop novel therapeutic products which will actually make a difference to the cancer patient, it must identify, nurture (and fund) more of his kind.

**S4**
**A NOVEL ROLE FOR BRCA1 IN MEDIATING THE INTERFERON GAMMA ANTIPROLIFERATIVE RESPONSE**
Heather Andrews[1#], Paul B. Mullan[1#], Stewart McWilliams[1], Sarka Sebelova[1], Jennifer E. Quinn[1], Paula M. Gilmore[1], Nuala McCabe[1], Amy Pace[2], Beverly Koller[2], Patrick G. Johnston,[1] Daniel A. Haber[3] and D. Paul Harkin[1*].

BRCA1 is a tumour suppressor gene implicated in the predisposition to early onset breast and ovarian cancer. We have generated cell lines with inducible expression of BRCA1 as a tool to identify downstream transcriptional targets that may be important mediators of BRCA1 function. Oligonucleotide array-based expression profiling identified twelve genes that were up regulated following inducible expression of BRCA1. Interestingly the majority of the identified targets were previously described interferon regulated genes. Northern blot analysis revealed that a subset of the identified targets including IRF-7, MxA and ISG-54 were synergistically up regulated by BRCA1 in the presence of interferon gamma but not interferon alpha or beta. Inducible expression of BRCA1 dramatically enhanced IFN-$\gamma$ mediated apoptotic cell death suggesting a functionally important role for BRCA1 in mediating the IFN-$\gamma$ antiproliferative response. In addition, a functional IFN-$\gamma$ pathway was found to be essential for BRCA1 mediated induction of IRF-7 and MxA. Conversely IFN-$\gamma$ mediated induction of IRF-7 and MxA was attenuated in the BRCA1 mutant cell line HCC1937. Finally reconstitution of the HCC1937 cell line with exogenous BRCA1 restored IFN-$\gamma$ mediated induction of IRF-7 and MxA in these cells. This study identifies BRCA1 as a component of the IFN-$\gamma$ regulated signalling pathway and suggest that BRCA1 may play a role in the regulation of the IFN-$\gamma$ mediated tumour surveillance pathway.

**S5**
**MARKERS AND MECHANISMS OF ENDOCRINE SENSITIVITY/RESISTANCE**
Professor Mitch Dowsett
Dept. of Biochemistry, Royal Marsden Hospital and Institute of Cancer Research, London SW3 6JJ

A number of hormonal therapies are available for the treatment of breast cancer each of which works either by reducing the synthesis of oestrogen or by antagonising oestrogen at the oestrogen receptor (ER) in breast carcinomas. Reduced oestrogen synthesis is achieved in pre-menopausal women by the ablation of ovarian function. This is achieved medically by the application of GnRH agonists. In postmenopausal women GnRH agonists are largely ineffective but aromatase inhibitors are an efficacious means of treatment. The ER antagonist tamoxifen also has a degree of oestrogen agonist activity which varies between tissues and which may limit its therapeutic efficacy. This mode of action has led to this type of drug being renamed a SERM (selective estrogen receptor modulator).

© 2002 Cancer Research UK

Oestrogens appear to exert their proliferative effects through ERα in breast carcinomas. ER negativity is the most widely used and reliable marker of de novo endocrine resistance. Of the 80% of patients that are ER positive most but not all will show some evidence of response to therapy. Progesterone receptor is an oestrogen-dependent protein and absence of this in ER positive tumours indicates a lesser likelihood of response but such tumours have a sufficiently good chance of response that this is not a useful indicator of resistance. EGF receptor and HER-2 appear to lead to a degree of resistance which may be dependent on the hormonal agent in use: benefit from tamoxifen may be reduced in patients that have ER positive HER-2 positive tumours and in model systems in which MCF7 human breast cancer cells have been transfected with HER-2. In contrast these cells show continued dependence on oestrogen and indeed in some reports hypersensitivity to oestrogen. Consistent with this, a recent neoadjuvant (pre-surgical) comparative trial found that patients that were ER positive and either HER-2 positive or EGF receptor positive had a far superior response to letrozole than to tamoxifen. Phosphorylation of ER by kinases downstream of these growth factor receptors appears to lead to increase sensitivity to the agonist effects of tamoxifen and to oestrogen stimulation which results in tamoxifen resistance but hypersensitivity to oestrogen deprivation.
Mechanisms of acquired resistance to oestrogen deprivation have been investigated to a lesser degree than tamoxifen. The major mechanism may be by acquired hypersensitivity to the residual levels of oestrogen which occur during treatment. Thus acquired resistance to GnRH agonist in premenopausal women may be followed by a second response on the addition of an aromatase inhibitor. These data are recapitulated by in vitro studies in which oestrogen deprivation leads to cells which are sensitive to stimulation by dosages less than $10^{-13}$M. Mechanisms for this have not been clearly delineated but enhanced activity of MAP kinase has been observed and differential activation of co-activators may also have influence.

### S6
### KEY ASPECTS OF SURGERY

Professor David George, Western Infirmary, Glasgow

ABSTRACT NOT RECEIVED

### S7
### RADIOTHERAPY: CURE AND CO-MORBIDITY

John Yarnold, Royal Marsden Hospital, Downs Road, Sutton SM2 5PT

The systematic overview of radiotherapy effects in women with early breast cancer published by the Early Breast Cancer Trials Collaborative Group in May 2000 announced a qualitative change in the rationale of radiotherapy in women with early breast cancer. For every 20 isolated local recurrences prevented by radiotherapy (&/or by surgery, presumably), there are 5 additional women alive at 10 years who would otherwise die of breast cancer. The only way a local-regional modality can achieve this effect is by the prevention of distant metastasis, the dominant cause of breast cancer mortality. Assuming cardiac shielding is confirmed by real-time electronic portal imaging during radiotherapy all patients, and assuming lymphatic radiotherapy is prescribed selectively, iatrogenic cardiac mortality will be eliminated and vascular mortality (stroke) minimised.
The greatest absolute mortality reduction is seen in axillary node positive patients, effects that are additive with the local and systemic effects of adjuvant systemic therapies. Eradication of axillary lymphatic metastases must be a determinant of cure in these patients (absolute reduction in 10-year mortality in node positive patients randomised to local-regional radiotherapy is 8 - 10%). The survival value of eradicating (by whatever modality) internal mammary chain metastases remains to be proven. One thing is certain; patients with occult internal mammary chain metastases (30% of axillary node positive women) currently have no prospects of cure.
Who should be prescribed radiotherapy? Patients with ≥ 10% risk of local recurrence should be offered high quality radiotherapy, thereby preventing 8 local recurrences and 2 breast cancer deaths at 10 years. This means all patients after breast conservation surgery, since there is not sufficient evidence for an identifiable group with a local recurrence risk of < 10% at 10 years. After mastectomy and optimal adjuvant systemic therapy, patients who are pN1-3+ also fall into this group, not just the particularly unfavourable pN4+ subgroup. Indeed, by restricting post-mastectomy radiotherapy to women with 4+ positive lymph nodes (as per the ASCO and St Galen consensus statements), there is a risk of throwing the baby (pN1-3+) out with the bath water, so to speak. Having eliminated the worst of old radiotherapy practices, and shed an unjustified nihilism with respect to local therapy, it is high time to implement the curative potential of modern high technology radiotherapy. Meanwhile, the UK lags behind other first World countries, with waiting lists of several months for curative radiotherapy. This is a crazy situation, which undermines the proven benefits of early diagnosis and rapid access to primary surgery. There can be no doubt that a minority of British patients lose their opportunity for cure whilst waiting for treatment.

### S8
### APOPTOSIS AND CARCINOGENESIS: ANALYSING THE CONSEQUENCES OF FAILURE TO ENGAGE DEATH PATHWAYS.

Alan R Clarke, Cardiff University, Cardiff, Wales, UK. CF10 3US

Loss of the apoptotic response has been implicated in carcinogenesis as a consequence of increased survival of DNA-damage bearing cells. We have directly tested this inference using a series of animal models. We have previously shown apoptosis to be dependent upon both p53 and the mismatch repair (MMR) protein *Msh2*. We now show that following certain forms of damage loss of these pathways translates into increased clonogenic survival and increased mutation, but that this is damage and tissue type sensitive. We have also analysed mice deficient for *Mlh1*. Loss of this gene appears less detrimental to the apoptotic response when compared to *Msh2* deficiency, yet the consequences for mutation frequency and clonogenic survival are comparable. Finally, we analysed mice mutant for the methyl binding domain protein *Mbd4*, which has been shown to interact with *Mlh1* and function as a thymine glycosylase. Our analysis shows *Mbd4* to be essential for the normal apoptotic programme following a range of DNA damage and that *Mbd4* deficiency can confer increased clonogenic survival *in vivo*. We now also show that *Mbd4* deficiency accelerates intestinal tumour development and alters the mutation spectrum in mice heterozygous for the $Apc^{Min}$ allele. Taken together our results demonstrate that mutations within many genes can impact upon the ability to engage apoptosis, however disruption of these genes cannot always be directly translated into increased survival, mutation and cancer predisposition.

### S9
### THE EMERGING ROLE OF MOLECULAR IMAGING FOR EVALUATING CANCER THERAPY

Brian D. Ross, Alnawaz Rehemtulla, Thomas L. Chenevert & Bradford Moffat
University of Michigan School of Medicine, Center for Molecular Imaging, Ann Arbor, MI USA

The capabilities of magnetic resonance imaging (MRI) and spectroscopy (MRS), along with optical imaging techniques such as bioluminescence imaging (BLI) for the pre-clinical study of cancer has benefited greatly from advances in both hardware and software. These advances provide exciting opportunities to investigate anatomical, cellular and molecular events using imaging methodologies. These opportunities can provide new insights and important information related to established, traditional tumor models. Additionally, these advances can be applied to emerging therapeutic modalities such as cancer gene therapy thus bridging the gap between gene delivery, quantitative monitoring of gene expression and correlating this expression with therapeutic outcome.
An overview of the results that have been obtained using MRI/S for studying the rat 9L brain tumor model will be given. MR studies on this well-established brain tumor model, used for the past 35 years, have yielded a wealth of new and vital information. Through the use of MRI for monitoring 9L tumor growth and response to therapy, we have observed that the current understanding of the effectiveness of traditional chemotherapies such as BCNU have been overestimated using traditional methods such as animal survival and colony forming assays (using cells obtained from disaggregated from solid tumors). In addition, we have found that cellular changes occur

© 2002 Cancer Research UK

very early following therapeutic intervention and can be detected prior to tumor Regression using diffusion-weighted MRI. This approach may provide an important clinical opportunity for individualizing and modifying therapeutic protocols during the course of treatment which was recently translated into clinical studies.
The use of MRI/S for quantitative noninvasive evaluation of expression of a therapeutic transgene in experimental tumors will also be presented. In this example, stable tumor cell lines expressing the cdna encoding for cytosine deaminase (CD) from yeast were grown in animals. Conversion of the nontoxic prodrug 5FC to 5FU by CD and the subsequent conversion to other metabolites could be quantitated using MRS. Finally, BLI was applied to both monitoring tumor growth and response to therapy and for following *in vivo* gene delivery using an adenoviral vector.
In conclusion, the use of noninvasive imaging modalities for quantitation of therapeutic outcome, gene delivery and monitoring of cellular events is emerging as an vital component for translating therapeutic agents from the lab into animals and humans.

**S10**
**PLASMA EPSTEIN-BARR VIRUS (EBV) DNA AS A TOOL FOR POST-TREATMENT MONITORING OF EBV-ASSOCIATED MALIGNANCIES**
Y.M. Dennis Lo
Department of Chemical Pathology, The Chinese University of Hong Kong, Prince of Wales Hospital, Shatin, New Territories, Hong Kong

Recently, much interest has been focused on the presence of nucleic acids of tumoral origin in the plasma and serum of cancer patients. Our group has used real-time quantitative polymerase chain reaction (PCR) to detect Epstein-Barr virus (EBV) DNA in the plasma and serum of patients with EBV-associated malignancies. Thus, for nasopharyngeal carcinoma (NPC), EBV DNA has been detected in 96% of NPC patients. We have shown that circulating EBV DNA concentration is an independent prognostic marker for this cancer. Furthermore, plasma EBV DNA has been shown to provide a good correlation with clinical events following anti-neoplastic treatment and to be predictive of clinical relapse. Apart from NPC, we have also shown that EBV DNA can be found in the plasma or serum of patients with other EBV-associated malignancies, including Hodgkin's disease, NK cell lymphoma and gastric carcinoma. Serial analysis of samples collected from patients with EBV-positive lymphomas also indicates that plasma EBV DNA measurement offers a powerful tool for the monitoring and prognostication of some of these malignancies.

**S11**
**BIOCHIPS IN CANCER RESEARCH: BEYOND cDNA MICROARRAYS**
Dr Olli Kallioneimi, National Human Genmome Research Insitute, NIH, USA

ABSTRACT NOT RECEIVED

**S12**
**A TUMOUR SPECIFIC TARGETED RADIOTHERAPY/GENE THERAPY FOR THE TREATMENT OF MALIGNANT DISEASE**
* Dr M Boyd : Department of Radiation Oncology, Cancer Research UK Beatson Labs, Garscube Estate, Bearsden Glasgow, G61 1BD.

Radiotherapy is, after surgery, the most widely used form of cancer treatment but the main limitation of radiation treatment is damage to normal tissues. This may be overcome by targeted radiotherapy - the selective delivery to malignant deposits of cytotoxic radionuclides bound to tumour-seeking agents. We are developing gene transfer techniques, which will allow malignant cells to be made more sensitive to radiation or cause them to take up radioactive drugs - providing tumour cell kill with reduced damage to normal tissues.
One of the most favourable targeting agents is [$^{131}$I]-labelled meta-iodobenzylguanidine ([$^{131}$I]MIBG), which is selectively concentrated in neuroblastoma cells bearing the membrane-bound noradrenaline transporter (NAT). In an attempt to apply [$^{131}$I]MIBG treatment to a range of tumour types, we recently introduced the NAT gene into UVW glioma cells, which previously did not express NAT. This resulted in active uptake of [$^{131}$I]MIBG and cell kill in a dose dependent manner (Boyd et al, 1999, 2001a). Subsequent studies have indicated that this gene therapy/targeted radiotherapy approach may be applicable to the treatment of a range of cancers.
No strategy currently exists for the introduction of transgenes into every cell of a tumour *in vivo*. Therefore approaches to cancer gene therapy must incorporate a component of collateral damage to non-transfected cells. A major advantage of our scheme is the inherent bystander effect afforded by radiation cross-fire to non-targeted cells from cells (NAT-expressing) which have taken up [$^{131}$I]MIBG. Having developed a novel three-dimensional culture system (transfectant mosaic spheroids - TMS) for characterisation of the extent of radiation-mediated bystander effects, we showed that when only 5% of a spheroid mass expresses the NAT transgene, the entire spheroid is susceptible to sterilisation by [$^{131}$I]MIBG. Recent experiments conducted with Professor Michael Zalutsky, Duke University, indicate that the same effect can be achieved using a 700-fold lower dose of the alpha-particle emitter [$^{211}$At]-astatine conjugated to benzylguanidine (i.e. [$^{211}$At]MABG).
In order to limit NAT transgene expression to tumour cells, we have employed, as transcriptional regulators, the human telomerase RNA (hTR) and protein component promoters (hTERT) (Boyd et al, 2001b). We are also investigating a range of gene delivery vehicles to facilitate clinical application of this therapeutic scheme. One such promising vector is the herpes simplex viral variant HSV1716. This could allow tumour cell kill by lysis and, following transgene delivery, targeted radiotherapy. *In vitro* experiments indicate enhanced cytotoxicity to human glioma cells using this two-hit scheme
In summary, this programme of research offers exciting alternatives to conventional radiation-based therapies and has the potential for treatment of a variety of human malignancies.
Boyd M, *et al.,* (1999). *Gene Therapy,* 6:1147; Boyd M *et al.,* (2001a). *J Gene Medicine* 3: 165; Boyd M *et al.,* (2001b) *Oncogene* 20:7804.

**S13**
**DESIGN AND EVALUATION OF ANTISENSE OLIGONUCLEOTIDES TARGETED AGAINST KIRSTEN *RAS* IN COLON ADENOCARCINOMA CELL LINES**
Paul J Ross*
CRC Centre for Cancer Therapeutics, Institute for Cancer Research and Gastroinestinal Unit, Royal Marsden Hospital, Downs Road, Sutton, SM2 5PT.

*Ki-ras* mutations are frequent in colorectal cancers a codon 12 valine mutation is the only specific mutation associated with an increased risk of recurrence and death. Antisense oligonucleotide therapy has the potential to selectively reduce expression of target genes. Therefore, the valine codon 12 Ki-*ras* mutation was targeted with a series of complimentary oligonucleotides. In a ribonuclease H cleavage assay 5 of a series of 7 oligonucleotides, cleaved the target RNA with variable efficacy and selectivity. However, none reduced *Ki-ras* mRNA or protein expression in the SW480 colon cancer cell line with the target mutation. Furthermore, a ribozyme targeted against this mutation was weakly active in a cell free assay, but had no effect on *Ki-ras* mRNA expression in SW480 cell lines. Computer modelling suggested that the valine codon 12 Ki-*ras* mutation was inaccessible to oligonucleotide hybridisation. Therefore, an antisense *Ki-ras* oligonucleotide was rationally selected by mapping cleavage sites on *Ki-ras* exons 1-3 mRNA incubated with a library of random sequence 17-mer oligonucleotides and *E. coli* RNase H. Three highly accessible regions of *Ki-ras* mRNA to oligonucleotides were identified. *Ki-ras* mRNA expression was variably reduced in the SW480 cell line after transfection with 11 oligonucleotides complimentary to accessible sites. The most effective oligonucleotide, KR4, reduced *Ki-ras* mRNA >90% 24 hours after treatment, and inhibited Ki-Ras protein expression 48 hours after treatment. Neither cell proliferation, nor cell cycle distribution was significantly different to controls. Vascular endothelial growth factor secretion was reduced to 56% of a control oligonucleotide ($p<0.001$). KR4 inhibition of Ki-Ras expression

© 2002 Cancer Research UK

inhibited phosphorylation of Erk 1/2 but not c-Akt phosphorylation. Expression profiling by gene array revealed altered expression of a number of genes following inhibition of *Ki-ras* expression.
KR4 inhibition of *Ki-ras* in SW480 cells affects factors influencing proliferation, vascularisation and metastasis. This justifies further studies on the effect of specifically inhibiting Ki-*ras* in spheroid growth models and *in vivo*.

**S14**
**STI571: A TYROSINE KINASE INHIBITOR FOR THE TREATMENT OF CML – VALIDATING THE PROMISE OF MOLECULARLY TARGETED THERAPY**
Brian J Druker
Oregon Health & Science University, Portland, Oregon USA

STI571 (imatinib mesylate, Gleevec, Glivec) is a rationally designed agent that inhibits the tyrosine kinase activity of Bcr-Abl, the causative molecular event in chronic myeloid leukemia (CML). STI571 has shown remarkable clinical benefits in all phase of the CML and the clinical results with STI571 will be reviewed. Issues related to clinical trials of molecularly targeted agents will be discussed including dose and patient selection. Other issues such as why STI571 has been so well tolerated and why cytogenetic responses occur will be explored. As relapses have occurred, most commonly in advanced phase patients, the mechanisms of relapse will be discussed along with possible means to circumvent resistance. STI571 not only inhibits the kinase activity of Bcr-Abl, but it also inhibits the c-kit and PDGF receptor tyrosine kinases. Other therapeutic targets for STI571, including gastrointestinal stromal tumors, which are caused by mutations that activate c-kit, will be discussed. These targets will be presented within a framework for deciding which tumors might be appropriate to treat with a molecularly targeted agent such as STI571.

**S15**
**PROGRESS AND CHALLENGES IN THE TREATMENT OF ESOPHAGEAL CANCER.**
Arlene A. Forastiere, Johns Hopkins University School of Medicine, Department of Oncology, Baltimore, Maryland, U.S.A.

Over the past two and a half decades there has occurred a striking change in the epidemiology of esophageal cancer with over a 350-fold increase in annual incidence rates of adenocarcinoma of the distal esophagus and gastro-esophageal junction in Caucasian males in the United States, Great Britain and Scandanavian countries. At the same time, clinical research efforts have resulted in combined modality treatment paradigms for both surgical and non-surgical management of esophageal cancer. Adenocarcinoma arising in the distal esophagus or straddling the G-E junction is distinct from squamous cell cancer of the esophagus in patterns of local-regional spread and failure but survival rates appear similar.
In the 1980s, a series of randomized, controlled trials were begun in the US evaluating the concurrent administration of chemotherapy and radiotherapy (RT) as definitive treatment for disease that had not overtly metastasized to distant sites. A landmark trial compared RT alone to RT plus 4 courses of cisplatin + 5-fluorouracil (5-FU) chemotherapy in patients with squamous cell cancer of the esophagus. A statistically significant improvement in median survival, 9 vs 14 mos. and actual 3-year survival, 0 vs 30% was demonstrated (Herskovic A et al. 1992; 326:1593). Subsequent efforts to improve outcome by intensifying chemotherapy or RT have shown added toxicities without therapeutic gain. Thus, 50 Gy RT and cisplatin + 5-FU is today the standard of care for patients with extensive local disease or medical conditions that preclude surgery.
The survival of patients undergoing surgery at major centers is 15-20%. This reflects the advanced stage (IIB or III) of most patients at diagnosis. To improve survival, several combined modality strategies have been investigated in randomized, controlled trials. These include: preoperative chemotherapy, and the combination of preoperative chemotherapy and RT. Preoperative cisplatin-based chemotherapy can substantially reduce tumor bulk in approximately 50% of patients and in about 5% there will be no tumor (path negative) left in the resected esophagus. Randomized trials comparing this strategy to surgery alone show conflicting results but the largest trial enrolling 800 patients in the UK showed a 10% survival improvement with preop chemotherapy. The concurrent administration of RT and chemotherapy prior to resection takes advantage of the radiation enhancing properties of cisplatin and 5-FU. Two-thirds of patients are downstaged by this treatment, 25-30% to path negative status. Three randomized trials of concurrent chemotherapy and RT followed by surgery compared to surgery alone have been reported (Walsh, Urba, Bossett ). Consistent findings for patients receiving preop chemo/RT in all studies were a 25-28% path negative rate and a 3-year survival rate of 32-36%. Two trials (Walsh, Urba) showed a survival advantage for patients receiving preop chemo/RT compared to surgery alone, 32% vs 6% and 32% vs 16%, respectively. These data support combined modality treatment for patients with locally advanced, resectable esophageal cancer.
The challenges ahead include understanding the progression of Barretts metaplasia to dysplasia and prevention of adenocarcinoma; and, adding molecularly targeted therapies to combined modality treatments to improve cure rates in patients with established squamous or adenocarcinoma of the esophagus and G-E junction.

**S16**
**THE FUTURE CONFIGURATION OF OESOPHAGEAL AND GASTRIC CANCER SERVICES - A SURGEON'S PERSPECTIVE**
Derek Alderson, University Division of Surgery, Bristol Royal Infirmary, Bristol BS2 8HW

NHS Executive guidance has indicated that improvement in outcome of patients with oesophageal and gastric cancer in the United Kingdom requires changes in service provision. The most significant change has been the creation of cancer networks, each covering approximately two million people. Within each network, it is suggested that specialist oesophagogastric teams are created with agreed protocols for patient care and that one or two teams within a network should function as cancer centres, at which all surgical resections will take place. The extent to which this might be achievable will be strongly influenced by resource allocation to deal specifically with these cancers.
The evidence that centralisation of surgical resources will improve outcomes is limited. Only a single United Kingdom study has carefully studied a large group of unselected patients and considered the influence of case mix. Decreased mortality was associated with an increased volume of operations/surgeon. The development of large multidisciplinary and multiprofessional specialist teams may be difficult to achieve and the evolution of surgical practice may still dictate that improved results can be obtained with population bases lower than those suggested by the NHS Executive. Reduced working hours for all surgical grades will also have a major impact, leading to larger clinical teams and greater concentration of care. On the basis of a minimum number of about 16 - 20 resections/surgeon/year to achieve and maintain competence, oesophagogastric cancer centres will require at least four surgeons. Concerns that operations hitherto carried out in many hospitals could lead to a loss of surgical skills and other resources at these institutions, remain unresolved. While some changes are inevitable, high quality contemporary audit remains fundamental to dictate the speed and extent of change.

**S17**
**WHO DOES WHAT, WHERE AND TO WHOM: EVIDENCE FROM THE SCOTTISH AUDIT**
Alastair Thompson, Dept of Surgery & Molecular Oncology, University of Dundee, UK

The Scottish Audit of Gastric and Oesophageal Cancer (SAGOC), the largest population based audit of these cancers ever conducted, identified 3293 patients with cancer of the stomach or oesophagus diagnosed in Scotland over a 2 year period.
The 3293 patients presented to 53 different hospitals; only a third of patients presented initially to a cancer centre. 33% of patients had a delay in presentation of >4 months. Endoscopy and biopsy was the primary method of diagnosis (in 94% of patients); patients presenting to smaller hospitals had

© 2002 Cancer Research UK

significantly less delay in establishing the diagnosis but 22% of patients waited > 4 weeks from GP referral. Staging was predominantly performed using CT scanning (in 69% of patients), ultrasound (30%) and laparoscopy (20%); those staged using CT with endoscopy or laparoscopy had the best outcome.

1302 patients (39.5%) were treated surgically, 1006 (30.6%) by surgical resection, with a 6.5% anastomotic leak rate and 12.9% post-operative mortality similar to single centre series reported from the UK. There were no statistically significant differences in operation rates, anastomotic leak rates, 30 day mortality or post operative mortality by size of hospital.

817 (24.8%) patients received chemotherapy and/or radiotherapy, with significant variation by health board ranging from 11% to 36%. 74% of these patients waited > 4 weeks for radiotherapy and 58% > 4 weeks for chemotherapy. Although toxicity was poorly recorded, 30% of patients had grade 3 or 4 toxicity and 3.9% died during therapy. There were significant differences by health board in the delivery of chemotherapy/radiotherapy and entry into clinical trials. 948 (28.8%) patients had endoscopic palliative therapy with significant variation by health board as to whether stent or laser was used; 11.6% of these patients had adverse events and 17% were alive at 1 year.

Overall the survival for gastric and oesophageal cancer was 32% (1 year) and 17% (2 years); patients undergoing surgery had survival of 54% and 33% respectively. Age, co-morbidity, level of physical activity, degree of dysphagia, pre-treatment aim of therapy and resection margin involvement were the major factors affecting survival.

SAGOC has demonstrated the extent of the cancer burden, variations in practice and adverse outcomes in Scotland. Sustained fiscal, personnel and organisational resource is urgently required to facilitate appropriate guideline based management and ongoing audit of an agreed minimum data set for these common cancers if improvements in survival are to be achieved.

### S18
### PREOPERATIVE CHEMOTHERAPY IN OESOPHAGEAL AND GASTRIC CANCER

David Cunningham, Royal Marsden NHS Trust, Surrey, UK

The outlook for the small proportion of patients with oesophageal or gastric cancer who present with resectable disease is disappointingly poor. 5-year survival figures following resection undertaken with curative intent range from 20-40%. Oesophago-gastric cancer is relatively sensitive to chemotherapy with response rates of 30-40% seen in patients with advanced disease. The addition of chemotherapy to surgery, with the intention of eliminating microscopic metastatic disease, would appear to be a worthwhile strategy to improve survival in this group. Moreover, there are potential advantages to preoperative administration. From the practical viewpoint, patients are better able to tolerate treatment, surgery may be made easier by tumour downsizing and the eradication of distant micrometastatic disease is likely to be more successful if dealt with at the earliest possible opportunity.

There are few large randomised studies looking at the role of pre-operative chemotherapy in this setting. A US multi-centre intergroup study randomised 440 patients to 3 pre-operative and 2 post-operative cycles of cisplatin and 5-fluorouracil (5-FU) or surgery alone, with no significant difference in survival between the 2 groups. Data from the MRC OE0-2 trial, which used a similar drug regimen but limited chemotherapy to the pre-operative setting, have been recently presented. 802 patients were randomised to surgery alone or 2 cycles of pre-operative cisplatin and 5-FU. Overall survival was significantly improved in the chemotherapy arm, with 2-year survival figures of 45% for chemotherapy and 35% for surgery alone. 87% of chemotherapy patients in this trial received 2 cycles of pre-operative therapy, and post-operative morbidity and mortality rates were not worse in this group. In the UK, pre-operative chemotherapy is now regarded as the standard of care for patients with operable oesophageal cancer.

Randomised trials of neo-adjuvant chemotherapy in operable gastric cancer have tended to be small, and have yielded conflicting data. A large MRC trial – the MAGIC trial – randomises patients to surgery alone or surgery plus 3 cycles of pre-operative and 3 cycles of post-operative ECF (epirubicin, cisplatin and infusional 5-FU: one of the most active regimens in advanced disease). Accrual is now complete and analysis of the data should provide valuable information to help guide future practice.

### S19
### GENE-ENVIRONMENT INTERACTIONS IN GASTRIC CANCER

Professor Emad M El-Omar, Department of Medicine & Therapeutics, Aberdeen University, Aberdeen, Scotland

*Helicobacter pylori* infection is associated with divergent clinical outcomes that range from simple asymptomatic gastritis to more serious conditions such as peptic ulcer disease and gastric cancer. The key determinants of these outcomes are the severity and distribution of the *H. pylori*-induced gastritis. Cancer occurs in a gastric milieu characterised by severe inflammation, hypochlorhydria and atrophy, all of which precede malignant transformation by decades. Host genetic factors are emerging as key determinants of disease risk for many cancers. Identifying candidate genes is a major challenge that has to stem from a profound understanding of the pathophysiology of the disease. In the case of *H. pylori*-induced gastric cancer, we speculated that the candidate genes would influence both gastric physiology and the inflammatory response to the infection. We initially targeted the pro-inflammatory cytokines interleukin-1β (IL-1β) and tumour necrosis factor-α (TNFα), both of which are inhibitors of acid secretion and key mediators of the host's response to the infection. We reported that pro-inflammatory polymorphisms in the *IL-1* gene cluster and the TNF-α gene (*TNF-A*) increase the risk of gastric cancer and its precursors in Caucasian populations. Our findings have since been expanded to include other candidate genes such as *IL-10*, and confirmed independently in other Caucasian and non-Caucasian populations. In summary, infected subjects with a pro-inflammatory host genetic makeup have an increased risk of developing severe gastritis, progressive gastric atrophy, hypochlorhydria and finally gastric cancer. Other gene-environment interactions clearly influence the rate of progression of these lesions and the ultimate malignant transformation.

### S20
### HISTONE MODIFICATIONS IN TRANSCRIPTIONAL CONTROL.

Helena Santos Rosa, Sylvain Daujat, Uta-Maria Bauer, Robert Schneider, Andy Bannister, Francois Fuks, Paul Hurd and Tony Kouzarides.
Wellcome/Cancer Research UK Institute, University of Cambridge, Tennis Court Road, Cambridge, CB2 1QR, UK.

Histone modifications are able to regulate transcription positively and negatively. Our ongoing analysis of the mechanism by which such modifications regulate transcription has focused on lysine and arginine methylation. Three distinct sites of methylation on histone H3 will be discussed, involving residues K4, K9 and R17.

Methylation at K9 is a repressive event for transcription at both heterochromatic sites and at promoters regulated by the RB repressor protein. We can now show that K9 methylation is involved in the repression of DNA methylated promoters by MeCP2. A K9 H3 methyltransferase associates with MeCP2 and is delivered to the differentially methylated domain of the H19 promoter.

Methylation at K4 results in transcriptional activation. Using antibodies raised specifically against the di- or tri-methylated state of K4, we show that only tri-methylated K4 correlates with activation of transcription in yeast. This result demonstrates a new rule for histone modifications in which the methyl state of a lysine is a consideration for activity. Given that any lysine can be mono-, di- or tri-methylated, the complexity of the code on histones is clearly much larger than previously suspected.

Methylation at R17 correlates with activity of estrogen regulated genes such as pS2. Using chromatin immunoprecipitations we have investigated the ordered appearance of modifications on the pS2 promoter following estrogen stimulation. We can show that acetylation by CBP takes place before methylation of R17 by CARM1 and that an acetylated H3 substrate augments methylation by CARM1. These results provide evidence for a "cross-talk" between CBP acetylation and CARM1 methylation.

© 2002 Cancer Research UK

**S21**
**TRANSCRIPTION THERAPY FOR CANCER**
Pier Paolo Pandolfi
Sloan-Kettering Institute, Memorial Sloan-Kettering Cancer Center.

Acute promyelocytic leukemia (APL) is characterized by the expansion of malignant myeloid cells blocked at the promyelocytic stage of hemopoietic development and is invariably associated with reciprocal chromosomal translocations always involving Retinoic Acid Receptor α (RARα) gene on chromosome 17. RAR variably fuses to PML, PLZF, NPM, NuMA and Stat5B genes (hereafter referred to as X genes/proteins). These translocations are balanced and reciprocal, thus leading to the generation of X-RAR and RAR-X fusion genes whose products coexist in the APL blast. The invariable involvement in these translocations of RAR, a prototypical transcription factor, makes APL a compelling example of aberrant transcriptional mechanisms in the etiopathogenesis of cancer. Here we will discuss the recent progresses made in defining the transcriptional basis underlying APL pathogenesis and role that the aberrant transcriptional activities of X-RAR and RAR-X fusion proteins play in leukemogenesis, reviewing the relevant therapeutic implications resulting from this analysis. We will address how this new understanding has allowed for the proposal and development of novel therapeutic strategies with transcriptionally active compounds such as histone deacetylase inhibitors (HDACIs), polyamide synthetic transcriptional regulators, and synthetic zinc-finger peptides, which are currently being tested in murine leukemia models and in human APL as well as in other tumor types.

**S22**
**THE MDR1 GENE: TRANSCRIPTIONAL ACTIVATION AND PHARMACOLOGICAL INTERVENTION**
K.W. Scotto Fox Chase Cancer Center Philadelphia PA

Although the basis for anti-cancer drug resistance is multifactorial, the overexpression of P-glycoprotein (Pgp), a membrane protein encoded in human cells by the MDR1 gene, has been causally linked to the multidrug resistant phenotype in a variety of experimental and patient tumors. Long known to confer resistance by mediating the efflux of drugs from the cell, more recent studies suggest that overexpression of P-glycoprotein also plays a general anti-apoptotic role that extends beyond resistance to chemotherapeutics, since cells which overexpress P-glycoprotein are resistant to a wide range of caspase-dependent apoptotic inducers, including serum starvation, Fas ligand ligation, UV-irradiation and tumor necrosis factor (Johnstone RW et. al Cell. 108:153, 2002).
It has been well established that *MDR1* gene expression can be activated by a variety of stressful stimuli. Studies in our laboratory indicate that the signals from these divergent stimuli converge on a region of the *MDR1* promoter that we refer to as the *MDR1* enhancesome (Jin, S and Scotto, K.W.. Mol Cell Biol. 18:4377, 1998; Hu, Z et al. J. Biol. Chem**.** 275:2979, 2000). This region includes binding sites for the trimeric transcription factor NF-Y and the Sp family of GC-binding factors. Together, these DNA-binding proteins recruit the histone acetyltransferase PCAF to the *MDR1* promoter, resulting in the acetylation of promoter-proximal histones and subsequent transcriptional activation that is likely mediated by further chromatin remodeling. The potential clinical relevance of this mechanism lies in the observation by our laboratory and others that expression of MDR1 is rapidly induced in human tumors during the course of chemotherapy. Thus, the role of the *MDR1* enhancesome in the regulation of transcription by a variety of stimuli makes it an attractive target for therapeutic intervention.
Ecteinascidin 743 (ET-743) is a tetrahydroisoquinoline isolated from the marine organism *Ecteinascidia turbinata* and presently entering phase III clinical trials in the US and Europe. We have previously shown that ET-743 interferes with the activation of *MDR1* transcription by multiple inducers, with minimal effect on constitutive *MDR1* expression even after a twenty-four hour exposure (Jin, S., et.al. Proc. Natl Acad Sci**.** 97: 6775, 2000). Our studies indicate that ET-743 exerts its effects through the stress-responsive *MDR1* enhancesome complex. Although the precise mechanism has yet to be elucidated, ET-743 does not interfere with the binding of NF-Y or Sp1 to the *MDR1* promoter, and thus would appear to act subsequent to complex binding and promoter-associated chromatin acetylation. Moreover, the effects of ET-743 are not restricted to a single class of DNA binding proteins. Indeed, ET-743 targets the transcriptional activation of a subset of promoters, including those for genes that are activated by the histone deacetylase inhibitor TSA. Our current working hypothesis is that ET-743 is a potent and novel inhibitor of activated but not constitutive transcription.

**S23**
**TRANSLATING CANCER EPIGENETICS INTO CANCER THERAPIES**
Robert Brown, Cancer Research UK Dept Medical Oncology, Beatson Laboratories, Glasgow University, Glasgow, G61 1BD, UK.
(r.brown@beatson.gla.ac.uk).

Epigenetic inactivation of genes is as important a driving force as genetic inactivation by gene mutation during tumorigenesis. Epigenetic transcriptional repression of tumour suppressor genes, cell-cycle genes, DNA repair genes and genes involved in invasion and metastasis, have been demonstrated in a wide variety of tumour types. Hypermethylated CpG islands, often associated with epigenetic silencing, may serve as potential markers for diagnosis and prognosis of cancer. CpG methylation of DNA can readily be analyzed using methylation specific PCR of candidate genes or more global approaches, such as differential methylation hybridization using CpG-island microarrays. Methylation patterns of tumour DNA identify patient groups with distinct methylation profiles and different clinical characteristics.
There is increasing interest in epigenetic regulation of gene expression for new approaches to cancer treatment. For many of the epigenetically silenced genes it has been shown that re-expression in tumour cells can lead to suppression of cell growth or altered sensitivity to existing anti-cancer therapies. Compounds have been identified that can readily reverse epigenetic silencing; for instance DNA methyltransferase (DNMT) and histone acetyltransferase (HDAC) inhibitors are currently undergoing clinical evaluation. Preclinical studies suggest that the combination of DNMT and HDAC inhibitors, together with existing therapies, holds particular promise. Pharmacodynamic measures of DNA methylation and histone acetylation, as well as subsequent effects on gene expression, will help to drive the appropriate scheduling of these combinations. Thus the combination of epigenomics, to identify those patients who will particularly benefit from these types of treatments, together with epigenetic pharmacodynamics will allow the full potential of these novel approaches to cancer treatment to be examined.

**S24**
**HISTONE DEACETYLASE AS A TARGET FOR CANCER THERAPY**
Nicholas La Thangue[*†], Robert Brown[‡], Paul Finn and Robert Williams[†],
*Division of Biochemistry and Molecular Biology, Institute of Biomedical and Life Sciences, Davidson Building, University of Glasgow, Glasgow G12 8QQ. Scotland UK. [‡]Department of Medical Oncology, CR UK Beatson Laboratories, Garscube Estate, Switchback Road, Glasgow G61 1BD, Scotland UK. and *[†]Prolifix Ltd, 91 Milton Park, Abingdon, Oxfordshire, OX14 4RY. England UK.

In eukaryotic cells the level of histone acetylation plays a vital role in chromatin remodelling and the regulation of gene expression. Histone deacetylases (HDACs) are a family of enzymes modulate histone acetylation, and the inhibition of HDAC activity has attracted considerable interest as a new therapeutic approach for cancer therapy. Low molecular target weight molecules such as trichostatin A (TSA) oxamflatin and suberoylanilide hydroxamic acid (SAHA) are potent HDAC inhibitors that block the growth of tumour cells.
Novel chemical series of low molecular weight and synthetically accessible inhibitors of HDAC have been designed, and assessed for activity in biochemical and tumour cell proliferation assays. A clinical development candidate, PXD101, which inhibits tumour growth by inducing apoptosis, was selected for further analysis. PXD101 blocks the growth *in vivo* of tumour xenografts in mice, with little apparent toxicity. Most interestingly, PXD101 exhibits marked activity in tumour xenografts of cisplatin resistant ovarian and tumour cell lines. Ongoing studies are focussed on understanding the spectrum of genes regulated by PXD101 and gaining further insights on the sensitivity of tumour cells to HDAC inhibition. Our

© 2002 Cancer Research UK

studies suggest that PXD101 has potential as a novel cancer therapeutic, and in this respect is being rapidly progressed as a viable candidate for clinical trials.

**S25**

**CANCER SERVICES LEAD THE MODERNISATION OF THE NHS**

Anna Gregor
Edinburgh Cancer Centre, Edinburgh ,UK

Over 50 years on - the NHS needs modernising. Cancer services have been in the fore- front of interest from public and politicians and clinicians and managers are taking the opportunities to make fundamental changes to the design, delivery and monitoring of clinical services in the UK. Many of the driving forces are shared across health care systems and not specific to cancer services. It is because of this generalisibility and because I know them best, that I will use examples from the Scottish model. .Survival outcomes have lagged behind most of our Europeans counterparts[1] and the differences are real as are the different resource bases ( financial, technological and availability of professional staff).It is tempting to associate these directly in a causative manner. UK has insufficient specialist capacity and poor scheduling which cause prolongation of treatment episodes, anxiety for patients and may worsen outcome. Fragmented service provision, lack of long term strategic planning and political targets distorting clinical priorities have led to services developing in a piecemeal and opportunistic way with resultant inequalities in provision and access. The political risk this represents has brought much needed investment into the NHS and cancer services have been major beneficiaries with the caveat that tangible improvements are expected in return.

So what needs to be done to deliver these? To catch up to EU average financially may just be possible over the next decade, but shortages of skilled manpower, the huge gap in technology infrastructure and the consequences of building programme will make this challenging and may not work with current models of care .The search for better model has brought: managed networks [2], audit and CSBS [3] and service redesign methodology from industry [4]. Multidisciplinary working forces examination of roles and responsibilities leading to expanding roles and changing functions [5]. Technology, particularly computerisation should facilitate cross organisational working but the capacity of the NHSS is lagging behind other developments. Fundamental to all of this is the central role of the patient as an active partner and the quality framework. By putting patients at the centre and devolving power over details of decision making to those responsible for service delivery we will improve experiences and outcomes and be able to share learning experiences with other parts of the NHS involved in chronic disease management.

EUROCARE-2, IARC No 151,1999
MEL (10), 1999
CSBS reports 2001
Kerr et al BMJ 2002
Richardson et al HEALTH policy 45 (2):119-132; 1998

**S26**

**FROM SCALPEL TO GENOME. SAVING LIVES IN COLORECTAL CANCER.**

Phil Quirke, Academic Unit of Pathology, University of Leeds.

Can we improve the present 5 year survival in colorectal cancer? The answer is certainly yes and relatively cheaply if current knowledge is taken and applied through new initiatives in surgery and pathology. In the future molecular pathology offers much hope.

The evidence for the importance of a high quality total mesorectal excision for rectal cancer and obtaining a 1 mm clear pathological circumferential margin will be described and the need for the introduction of a quality assured national training program for surgeons, pathologists and radiologists discussed.

High quality pathological reporting is important. to identify node positive cases and high risk Dukes B cases and work to date on our UK clinical trials studies suggest that pathological factors are the most important factors for predicting a poor prognosis. The Royal College of Pathologists national minimum dataset should be used routinely as it improves the level of staging data available and when used in trials improves the yield of lymph nodes which in turn directs adjuvant therapy.

The translation of the knowledge of the sequencing of the human genome into practice offers huge challenges. Molecular pathological studies may identify new prognostic and predictive studies but they should be performed in the context of prospective randomized control trials with gold standard high quality histopathological data. Early studies in the UKCCCR AXIS study suggest that the preservation of a normal 18q may preserve responsiveness to 5FU therapy and that many of the currently fashionable markers (microsatellite instability or loss of expression of hMLH1) are not helpful for either prognosis or prediction of responsiveness to therapy.

New technologies are greatly increasing the power of such studies. cDNA arrays are suggesting many new targets for investigation, tissue microarrays of hundreds of tumours allow rapid evaluation of these putative markers and quantitation of antigen expression by immunocytochemistry and in situ hybridization is now feasible. The use of these technologies will be described in current UK colorectal trials. Programs for the organization and distribution of such material and standards for their analysis need to be developed to allow rapid assessment of the many putative markers that will emerge and their comparison to good pathological and clinical data.

The funding of a UK quality assured rectal cancer training program would improve outcomes in rectal cancer more quickly than at any time over the last 50 years. The improving quality of pathological reporting, the major advances in new technology and the centrally organized coordination of molecular pathology in clinical trials hold hope for further improvements in the future.

**S27**

**COMPUTATIONAL SIMULATION OF GROWTH FACTOR RECEPTOR CLUSTERING**

W.J.Gullick, J.P.Goldman C.G.Johnson[1] and N.L.V.Hayes
Cancer Biology Laboratory, Department of Biosciences, University of Kent at Canterbury, Canterbury, Kent CT2 7NJ and [1] Computing Laboratory, University of Kent at Canterbury, Canterbury, CT2 7NF

The Epidermal Growth Factor Receptor family consists of four receptors (EGFR, c-erbB-2, c-erbB-3 and c-erbB-4) and at least ten ligands. It has been proposed that some ligands act principally to form homodimeric receptor combinations and others to produce heterodimers. This system is involved in control of growth, differentiation, locomotion and death of normal cells and has been demonstrated to be one of the main causes of cell transformation in many common solid tumour types.

Much work on this system has used detergent solubilised cells but this approach lacks the ability to measure the position of the receptors, ligands and second messengers, a factor vital in understanding their interactions. We have taken two approaches to analysing the system while retaining this information. Firstly we have tagged the EGFR and c-erbB-2 receptors with green fluorescent protein and determined their life cycle individually in live cells or in combinations using fixed cells and immunofluorescent staining. This work has shown that the receptors apparently do not use the same mechanisms to reach the cell membrane nor are they always present in the same subcellular structures at the same time.

Second we have constructed a computational simulation of receptor interactions (see http://www.cs.ukc.ac.uk/people/staff/cgj/research/receptors.html) using object oriented modeling and have displayed the results using Java Graphics. In this system the strengths of receptor interactions and the degree of ligand occupancy can be varied. The results are displayed graphically or can be analysed numerically. This system can be used to determine receptor behaviour under different conditions and potentially to predict responses and interaction strengths.

The combination of these approaches should lead to a better understanding of the system and to identify sensitive interactions as targets for new inhibitory drugs.

© 2002 Cancer Research UK

S28
**TARGETS FOR TREATMENT: LESSONS FROM AIDS-RELATED MALIGNANCIES**
Chris Boshoff, Teenage Cancer Unit, and Cancer Research UK Viral Oncology Group, Wolfson Institute for Biomedical Research, University College London

Most cancers that are associated with HIV infection are driven by oncogenic viruses, such as Epstein-Barr virus (EBV), Kaposi's sarcoma-associated herpesvirus (KSHV) and human papillomavirus (HPV). Gaining insight into the epidemiology and mechanisms that underlie AIDS-related cancers has provided us with a better understanding of cancer immunity and viral oncogenesis.
The increased risk of cancer in immunosuppressed patients after organ transplantation, and in patients with inherited defects in immune function, such as severe combined immune deficiency, was thought to support the immune surveillance hypothesis. However, immunosuppressed individuals are not at an increased risk of developing some of the most common malignancies, such as breast, prostate or colon cancer. Most of the cancers that do develop during states of immunodeficiency are rare cancers associated with viral infection. In fact, the risk of developing the most common types of epithelial cancers has even been reported to be reduced in immunodeficient patients.
The molecular pathways exploited by the oncogenic viruses, enabling them to survive as intracellular parasites and complete successful replication of their genomes, are often pathways disrupted in non-viral associated malignancies. The cellular responses to viral entry and viral protein expression are similar to the cellular countermeasures after activation of protooncogenes, and include senescence and/or apoptosis. KSHV, EBV and HPV have evolved counterattacks to these cellular responses including blocking apoptosis by interfering with the functions of p53, FAS/CD95/FADD pathway and effector caspases; interfering with B-cell signalling (EBV and KSHV) by taking over the B-cell antigen-receptor (BCR) signalling pathways, and these viruses induce various signal transduction pathways, leading to cell proliferation.

S29
**ANTISENSE AND APOPTOSIS SENSITISATION FOR MALIGNANCY**
Finbarr E, Cotter. Dept Experimental Haematology, Bart's and the London School of Medicine, Turner St, London E2 1AD, UK. email*;*
*f.e.cotter@qmul.ac.uk*

Many cancer cells have an inherent resistance to chemotherapy due to the abnormal expression of anti-apoptosis genes, such as Bcl-2. The effect is mediated through the mitochondrium. The anti-apoptosis proteins maintain the the mitochondrial permeability transition pore complex PTPC, spanning the membrane, in the closed position, resulting in a block to release of pro-apototic factors. Antisense oligonucleotides (ASO) complementary to the mRNA have the ability to "switch off" gene expression and have been applied to patients with B-cell lymphomas. A phase I study using Bcl-2 ASO (G3139, Genta,USA) has been showing evidence of efficacy and acceptable toxicity. This study demonstrates evidence of down regulation of the target protein in humans by a sequence specific ASO effect. A similar study in chronic lymphatic leukaemia (CLL) has shown good efficacy with a dose of 3mg/kg/day and in vitro studies suggest that a combination with Rituxan (anti CD20) improves the response further. This has been followed by a phase II study combining Bcl-2 ASO with chemotherapy for a number of different types of malignancies including lymphoma, breast cancer, chronic lymphatic leukaemia (CLL) , acute myeloid leukaemia and malignant melanoma (with high levels of Bcl-2 protein). It has been confirmed that Bcl-2 ASO works through a true antisense mechanism and has the ability to "open" the PTPC. Bclxl, another anti-apoptosis gene, is also implicated in cancer chemoresistance. An effective ASO has been developed (ISIS, Carlsbad, USA) with both *in vitro* and *in vivo* efficacy. The Bcl-2 and Bclxl mediation of chemoresistance through the mitochondrial PTPC occurs in association with the mitochondrial benzodiazepine receptor. Small molecules have been developed that act as benzodiazepine receptor ligands, to overcome anti-apoptosis gene in a similar manner to Bcl-2 ASO to overcome chemoresistance. PK11195 is such a molecule. These act on the same pathway as the Bcl-2 and Bclxl ASOs and may provide additional therapeutic molecules. With care novel therapies based on the biology of the cancer cell may help improve the treatment of patients with malignancy.

S30
**PHARMACOLOGICAL RESTORATION AND PROMOTION OF P53 TUMOUR SUPPRESSOR ACTIVITY**
Barbara Foster and Farzan Rastinejad Pfizer Global Research and Development, Groton, CT USA

Disruption of the p53 tumor suppressor pathways in virtually all human cancers offers a unique opportunity to discover drugs that impact a broad range of tumor types. Drug-like small molecules have been identified that act on the isolated DNA binding domain of p53 to specifically promote stability of the active p53 conformation. These compounds can modulate p53 conformation and transcription activity in cells and in tumors and suppress the growth of tumor xenografts with naturally mutated p53. CP-31398, a prototype modulator of p53 conformation, can dramatically improve the proapoptotic effects of standard cancer chemotherapy drugs and Trail in tumor cells that harbour mutant p53. Recent data also suggest that CP-31398 not only restores the tumor suppressor activity of mutant p53, but that it can also promote p53 activity in tumor cells that harbour wild-type p53. Taken together, these findings suggest that the conformational stabilization of wild-type and mutant p53 represents an attractive opportunity to develop a single molecular-based therapy, that either alone or in combination with standard agents, could be used to treat the vast majority of human malignancies.

S31
**DT-DIAPHORASE AND PRODRUG ACTIVATION**
Shiuan Chen, Ph.D.
Div. of Immunology, Beckman Res. Institute of the City of Hope, Duarte CA, USA

DT-diaphorase, also referred to as NQO1 or NAD(P)H: quinone acceptor oxidoreductase, is a flavoprotein that reduces quinones and quinonoid compounds to hydroquinones, using either NADH or NADPH as the electron donor. It consists of two identical subunits. Each subunit has a molecular weight of 30,000 and contains one FAD prosthetic group, noncovalently attached to the protein. DT-diaphorase is thought to play an important role in protecting tissues against the mutagenic, carcinogenic and cytotoxic effects of semiquinones that occur widely in nature. DT-diaphorase catalyzes a two-electron reduction of quinones, without the formation of semiquinones. DT-diaphorase is also known to reductively activate cytotoxic anti-tumor quinones such as mitomycins, anthracyclines, and aziridinyl-benzoquinones, as well as nitrobenzamides such as CB 1954 [5-(aziridin-1-yl)-2,4-dinitro-benzamide]. Enzymatic reduction of these anti-tumor compounds gives rise to reactive intermediates that can then undergo nucleophilic additions with DNA and other macromolecules, suggesting a possible mechanism for their cytotoxicity. A natural mutation of DT-diaphorase, P187S, and an isoenzyme of the enzyme, NQO2 (dihydronicotinamide riboside-quinone oxidoreductase-2), have been identified. Their catalytic functions will be discussed and compared to those of DT-diaphorase.

S32
**NITROREDUCTION – A TUMOUR SELECTIVE PRODRUG ACTIVATION MECHANISM ?**
Richard Knox, Enact Pharma PLC, Porton Down, Salisbury, SP4 0JQ

CB 1954 [5-(aziridin-1-yl)-2,4-dinitrobenzamide] represents one of the very few examples of a compound that shows a real anti-tumour selectivity. Unfortunately, this is only seen in certain rat tumours. CB 1954 is a prodrug that is enzymatically activated to generate a difunctional agent, which can form DNA-DNA interstrand crosslinks. The bioactivation of CB 1954 in rat cells involves the aerobic reduction of its 4-nitro group to a 4-hydroxylamine by the enzyme NQO1 (DT-diaphorase). The human form of NQO1

© 2002 Cancer Research UK

metabolises CB 1954 much less efficiently than rat NQO1. Thus human tumours are insensitive to CB 1954.
In view of the proven success of CB 1954 in the rat system, it would be highly desirable to re-create its anti-tumour activity in man. This has led to the development of CB 1954 analogues and other prodrugs activated by nitroreduction such as those based on a self-immolative activation mechanism. A gene therapy-based approach for targeting cancer cells and making them sensitive to CB 1954 and related compounds has been proposed. GDEPT (gene-directed enzyme prodrug therapy) has been used to express an *E. coli* nitroreductase in tumour cells and human tumour cells transduced to express this enzyme are very sensitive to prodrugs activated by nitroreduction. CB 1954 is in clinical trial for this application.
Recently it has been show that a latent nitroreductase is present in some human tumours. This is NQO2 - an enzyme that requires for activity the non-biogenic compound dihydronicotinamide riboside (NRH) as a cosubstrate. When active, NQO2 is 3000 times more effective than human DT-diaphorase in the reduction of CB 1954. NRH and reduced pyridinium derivatives that, like NRH, act as co-substrates for NQO2 produce a dramatic increase in the cytotoxicity of CB 1954 against human cell lines *in vitro* and its anti-tumour activity against certain human xenografts *in vivo*. NQO2 activity is substantially raised in tumour samples from colorectal and hepatoma patients (up to 14-fold). A phase I clinical trial of an NQO2 co-substrate with CB 1954 is scheduled to start this year.

**S33**
**POLYMER THERAPEUTICS AND THEIR COMBINATIONS FOR TUMOUR-SPECIFIC DELIVERY**
Ruth Duncan, Centre for Polymer Therapeutics, Welsh School of Pharmacy, Cardiff University, King Edward VII Avenue, Cardiff CF10 3XF, UK

An increasing number of polymer-anticancer conjugates are entering Phase I/II clinical trials. Early results are very promising, especially as several of the polymeric carriers had never before been used in humans, and the optimum dosing schedule for such macromolecular prodrugs is still unknown. Consistently throughout these trials antitumour activity has been seen in chemotherapy refractory patients. The conjugates include; dextran-doxorubicin, HPMA copolymer-doxorubicin PK1, FCE28068), HPMA copolymer-doxorubicin-galactosamine (PK2, FCE 28069), HPMA copolymer-paclitaxel (PNU166945), HPMA copolymer-camptothecin (MAG-CPT), polyglutamate-paclitaxel (CT-2103), HPMA copolymer-platinate (Access Pharmaceuticals), and PEG-camptothecin (Enzon Inc). Three groups of compound have emerged. Conjugates that have failed because of toxicity of the carrier (dextran-doxorubicin); conjugates that have failed because of unacceptable toxicity of the conjugate (HPMA copolymer-paclitaxel and HPMA copolymer-camptothecin probably due to inadequacy of the polymer-drug linkage, and finally those conjugates that have progressed into further Phase I/II trials. Two interesting Phase I studies are currently ongoing with the PGA-paclitaxel conjugate either as a single agent or combination with cisplatin at a dose of 75 $mg/m^2$ - the first combinations to be tested in man.
So far, HPMA copolymer-doxorubicin-galactosamine designed to target liver and heptaocellular carcinoma via the asialoglycoprotein receptor is the only conjugate designed for receptor-mediated targeting. Most conjugates were designed to capitalise on passive tumour targeting by the EPR effect. In preclinical models this mechanism of targeting (due to hyperpermeable angiogenic vasculature) can give rise to 20 % dose/g in tumour tissue. Often the highest degree of targeting is seen in the smallest tumours giving hope for benefit in localisation in small micrometastases. Our current research is exploring several different therapeutic strategies that are designed to capitalise on improved tumour targeting by the EPR effect: (1) Lysosomotropic delivery Synthesis of conjugates containing novel, more potent drugs that also have novel mechanisms of action. (2) Intracytoplasmic delivery Use of bioresponsive polymers promote cytosolic access of genes and proteins. (3) Delivery of drug extracellullary Combination approaches such as polymer-directed enzyme prodrug therapy (PDEPT) or polymer-directed enzyme liposomal therapy (PELT), and (4) Membrane-active polymer conjugates: Use of polymers as a platform for targeted delivery of antitumour agents that are active at the level of the plasma membrane.
The current status of this field will be reviewed and future opportunities/challenges. addressed.

**S34**
**DESIGN OF TISSUE-SPECIFIC OLIGOPEPTIDE PRODRUGS**
D J Mincher, Anticancer Drug Design and Delivery Research Group, School of Life Sciences, Napier University, Edinburgh, EH10 5DT, UK.

The clinical usefulness of many low molecular mass antitumour compounds is restricted because of their narrow therapeutic index. Rational drug design of new cancer chemotherapeutics against identifiable molecular targets, that ideally should be causal factors in the pathogenesis of disease, has so far been relatively unproductive at effecting cures for most malignancies; this approach is hampered by currently incomplete identification of all the molecular targets responsible for the majority of these cancers.
An alternative approach is to capitalise on phenotypic rather than genetic differences between tumour cells and normal cycling tissues. Early examples of prodrugs of cytotoxins fail to meet the criteria for phenotypic targeting due to non-specific activation mechanisms, however, recent advances in the development of *tumour-activated prodrugs* have shown substantial improvements in antitumour activity; promising, non-macromolecular examples will be reviewed.
Synthetic organic-peptide conjugation chemistry is being applied to the design of tumour-activated prodrugs that transport and release the active agents in the tumour environment. The focus is the rational design of oligopeptide prodrug conjugates that are capable of harnessing 'the dark side' of endogenous protease activity to achieve tissue-specific drug delivery and improve therapeutic index.

**S35**
**THE ROLE OF CYP1B1 AS A TUMOUR SUPPRESSOR ENZYME**
G.A. Potter, Cancer Drug Discovery Group, School of Pharmacy, De Montfort University, Leicester LE1 9BH.

A case is presented for the enzyme CYP1B1 functioning as a tumour suppressor enzyme which acts via the bioactivation of natural dietary compounds into growth inhibitory substances. The enzyme CYP1B1 is a human cytochrome P450 enzyme belonging to the CYP1 family, which comprises three member CYP1A1, CYP1A2, and CYP1B1. CYP1 family enzymes are known to metabolise xenobiotics and to activate procarcinogens into active carcinogens. CYP1B1 is of interest since it is highly expressed in many different types of human tumour (e.g. breast, colon, lung) but is not expressed in the normal tissue. This has led some researchers to conclude that CYP1B1 is involved in the cause of tumours due to its carcinogen activating ability. However this is a very simplistic view and does not explain why the CYP1B1 enzyme should be present in tumours in the first place, nor how it can be expressed in such a wide varity of tumours irrespective of their different oncogenic origins. We have developed an alternative hypothesis whereby CYP1B1 is functioning in an opposite sense, being there to destroy the tumour cells. Here we propose that CYP1B1 functions as a tumour suppressor enzyme through natural prodrug bioactivation. Preliminary evidence for this hypothesis has now been obtained and natural prodrugs with cancer preventative properties have been identified.
[1]Murray, G.I., Taylor, M.C., McFadyen, M.C.E., McKay, J.A., Greenlee, W.F., Burke, M.D. & Melvin, W.T. *Cancer Res.*, 57, 3026-3031, 1997
[2]Potter, G.A., Patterson, L.H. & Burke, M.D. Aromatic hydroxylation activated (AHA) prodrugs. *US Patent 6,214,886*, 2001
[3]G.A. Potter, L.H. Patterson, E. Wanogho, P.J. Perry, P.C. Butler, T. Ijaz, K.C. Ruparelia, J.H. Lamb, P.B. Farmer, L.A. Stanley, and M.D. Burke, *Br. J. Cancer*, 86, 774-778, 2002.

© 2002 Cancer Research UK

